# A Wireless Monitoring System Using a Tunneling Sensor Array in a Smart Oral Appliance for Sleep Apnea Treatment

**DOI:** 10.3390/s17102358

**Published:** 2017-10-16

**Authors:** Kun-Ying Yeh, Chao-Chi Yeh, Chun-Chang Wu, Kuan Tang, Jyun-Yi Wu, Yun-Ting Chen, Ming-Xin Xu, Yunn-Jy Chen, Yao-Joe Yang, Shey-Shi Lu

**Affiliations:** 1 Graduate Institute of Electronics Engineering, National Taiwan University, Taipei 10617, Taiwan; d00943015@ntu.edu.tw (C.-C.W.); r04943121@ntu.edu.tw (K.T.); r05943114@ntu.edu.tw (J.-Y.W.); sslu@ntu.edu.tw (S.-S.L.); 2 Graduate Institute of Mechanical Engineering, National Taiwan University, Taipei 10617, Taiwan; scott@mems.me.ntu.edu.tw (C.-C.Y.); ytchen@mems.me.ntu.edu.tw (Y.-T.C.); xmx@mems.me.ntu.edu.tw (M.-X.X.); yjy@ntu.edu.tw (Y.-J.Y.); 3 Department of Dentistry, National Taiwan University Hospital, Taipei 10617, Taiwan; chenyj@ntu.edu.tw

**Keywords:** sleep apnea, OSA, smart oral appliance, SoC, sleep monitoring, tunneling sensor, wireless, prototype module

## Abstract

Sleep apnea is a serious sleep disorder, and the most common type is obstructive sleep apnea (OSA). Untreated OSA will cause lots of potential health problems. Oral appliance therapy is an effective and popular approach for OSA treatment, but making a perfect fit for each patient is time-consuming and decreases its efficiency considerably. This paper proposes a System-on-a-Chip (SoC) enabled sleep monitoring system in a smart oral appliance, which is capable of intelligently collecting the physiological data about tongue movement through the whole therapy. A tunneling sensor array with an ultra-high sensitivity is incorporated to accurately detect the subtle pressure from the tongue. When the device is placed on the wireless platform, the temporary stored data will be retrieved and wirelessly transmitted to personal computers and cloud storages. The battery will be recharged by harvesting external RF power from the platform. A compact prototype module, whose size is 4.5 × 2.5 × 0.9 cm^3^, is implemented and embedded inside the oral appliance to demonstrate the tongue movement detection in continuous time frames. The functions of this design are verified by the presented measurement results. This design aims to increase efficiency and make it a total solution for OSA treatment.

## 1. Introduction

Sleep quality is one of the most concerned medical topics. Lots of mental and physical causes will lead to poor sleep, and sleep apnea has the strongest connection with the physical structure among all the causes. Sleep apnea is a serious sleep disorder that occurs when a person’s breathing is interrupted during his sleep, even over 30 times an hour. The most common type (over ~80%) of sleep apnea is obstructive sleep apnea (OSA), which occurs when the throat muscles intermittently relax and block the airway during sleep [1]. More than 15% of the population (~50 million people) in US suffers from sleep-disordered breathing [2], and this number is still growing predictably. OSA is not a fatal disease, but the main influence is lowering the blood oxygen and cutting the whole sleep into pieces. The lack of oxygen will potentially result in many health problems, including high blood pressure, stroke, heart failure, diabetes, and depression [3]. Furthermore, untreated OSA also causes daytime sleepiness and increased risk of associated accidents (e.g., falling asleep while driving) [4].

Lots of approaches are available for OSA treatment, including nonsurgical options such as behavioral control (loss weight, stop smoking, and so on), continuous positive airway pressure (CPAP) and oral appliance for general OSA patients and surgical options such as Uvulopalatopharyngoplasty (UPPP), Nasal reconstruction, and Tracheostomy for severe OSA management. Oral appliance therapy is an effective treatment option [5] which is suitable to manage mild to moderate degree of OSA. Using the oral appliance is also popular with patients because it is quiet, low-cost, portable, and easy to wear and maintain. The most common mechanism of action is to hold the tongue or protrude the mandible in a more anterior position, which tightens the soft tissue and muscles of the upper airway. The proper position of the tongue and increased reflex activity of airway muscles will be helpful to prevent airway obstruction or collapse. Consequently many slight adjustments are required to fit the intraoral need of each patient. To improve its effectiveness, some researches combined the oral appliance with pressure sensors to detect tongue position [6,7,8] but didn’t make it a total solution. There are some other works about wearable health monitoring systems using soft sensing technologies to detect respiratory activities [9,10,11]. These systems with flexible sensors achieve real-time monitoring with promising results but need to incorporate with bulky equipment for subsequent signal processing. Thus they won’t be suitable for the portable and wireless applications. This paper presents an integrated system capable of continuous sleep monitoring in an oral appliance to intelligently position the tongue movement and connect with the cloud to enhance therapy effectiveness and efficiency.

The first challenge of system design is to detect the subtle pressure from the tongue movement (~70 kPa maximum [12,13,14]), which is difficult to be detected by typical pressure sensors. In this paper, a novel piezoresistive sensor array, whose conductivity is governed by tunneling effect [15], is fabricated and integrated with the prototype module to detect this subtle pressure. Using a contact surface of interlocked microdome structures, this tunneling sensor presents an ultra-high sensitivity comparing to those made of typical conductive polymer. Simple fabrication process, low cost, low power consumption, rapid dynamic response, good scalability, repeatability and flexibility are all its key features which make it a significant and unique role in this system.

Another challenge of system design is the very limited module size. Because the whole system with all the on-board components and sensor array will be integrated and embedded in an oral appliance, the module has to be sufficiently compact to fit in the available space. System-on-a-Chip (SoC) technology, which has characteristics of small size, multi-sensing, the capabilities of wireless communication and inductively charging, has been widely adopted to solve this problem in recent years [16,17]. All the function blocks required by the system can be specifically designed and merged on a single chip to optimize system performance and reduce the total size and power consumption. Power consumption is also seriously concerned, especially in a continuous sleep monitoring system of 8-straight-hour operation. Therefore, a specifically designed SoC with a power management unit (PMU) is employed on the prototype module for function demonstration.

The following section will introduce the proposed method and essential materials, including the tunneling sensor and SoC chip of this work. Next, the fabrication and measurement results are presented with discussions, followed by the conclusion.

## 2. Methods and Materials

### 2.1. System Application Scenario

**Figure 1:**
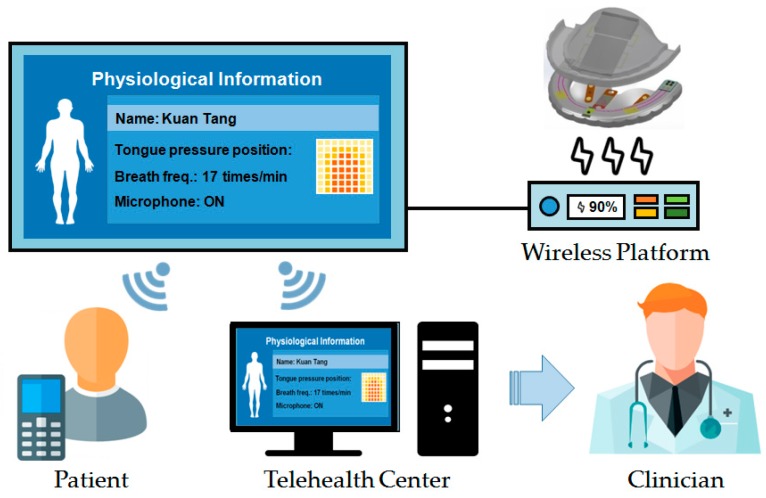
Application scenario of the proposed system.

Figure 1 shows application scenario of the proposed system [18]. Each night the OSA patient wears the proposed smart oral appliance during sleep (assuming an average of 8-h sleep). During the therapy, the intraoral device alleviates OSA symptoms and collects data automatically. After getting up, the patient places the device on the wireless platform which retrieves physiological data and transmits it to his personal computer and cloud storages. This allows the clinicians to monitor intraoral activities during the whole sleep and make subsequent diagnoses much explicit. Thus the proposed system strengthens the connection between the patients and clinicians and improves the therapy efficiency. The battery in the device is simultaneously recharged on the platform wirelessly to sustain the next usage.

### 2.2. Tunneling Piezoresistive Sensor

**Figure 2:**
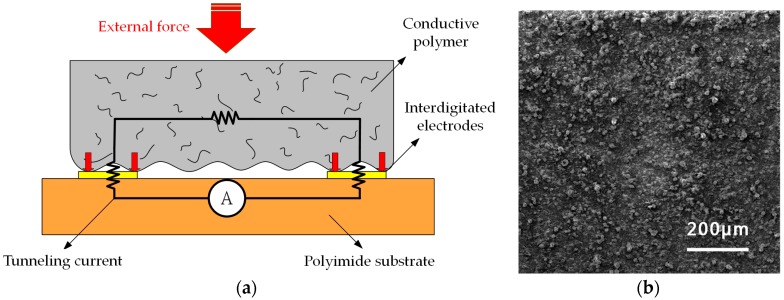
(**a**) Cross-sectional view and operational principle of the tunneling sensor; (**b**) SEM image of the contact surface of interlocked microdomes.

To precisely sense the subtle pressure from the tongue movement (typical: ~tens kPa, maximum: ~70 kPa [12,13,14]), a tunneling piezoresistive sensor with ultra-high sensitivity of −1.04 kPa^−1^ under tiny pressure (<3 kPa) is utilized [15]. Figure 2a illustrates the cross-sectional view and operational principle of this sensor. The device is composed of two layers: the upper conducive polymer layer with a contact surface of interlocked microdomes and the lower polyimide substrate layer with interdigitated electrodes. When the external pressure is induced, the contact area between interdigitated electrodes and microdome structures made of multiwalled carbon nanotubes and poly-dimethylsiloxane (MWCNT and PDMS) increases significantly, which in turn reduces the tunneling resistance sharply.

Figure 2b shows a SEM image of the contact surface of interlocked microdomes manufactured by the membrane filter. Such tunneling piezoresistive sensors with unique microdome structures have much higher sensitivity than those composed of typical conductive polymer materials, whose conductivities are dominated by the percolation theory [19,20,21]. This feature provides an excellent immunity against the variation of device fabrication and background noises from the integrated system. Furthermore, the microdome structure, which has instantaneous surface deformation from induced pressure in the interlocked geometry, leads to a much rapid response comparing to that of planar conductive composite films.

**Figure 3:**
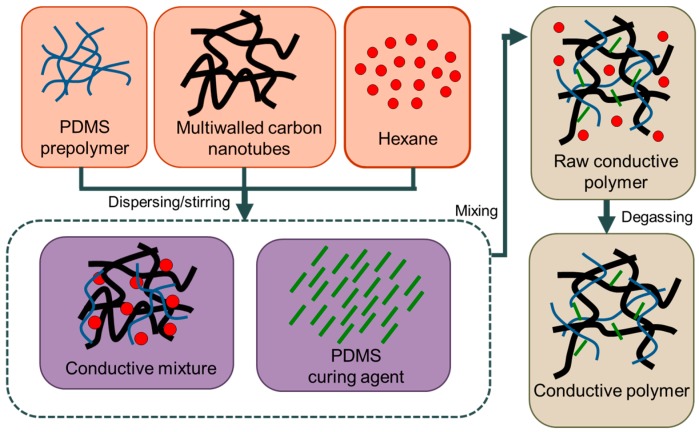
The preparation of conductive prepolymer used in the proposed sensor array.

The preparation of conductive prepolymer used in the proposed sensor array is shown in Figure 3. MWCNTs with diameters of approximately 20 nm and lengths ranging from 10 to 50 μm are distributed in hexane and PDMS prepolymer whose CNT concentration is 6 wt % in a ratio of 4:1. The mixture is thoroughly stirred using a magnetic stirrer for 2 h. The hexane is utilized as the dispersant for enhancing the fluidity of the PDMS prepolymer and improving the mixture uniformity during blending. A curing agent is subsequently mixed with the prepolymer in a 10:1 ratio, and the mixture is stirred for another 30 min. In the end, the MWCNT–PDMS prepolymer is degassed in a vacuum chamber for 30 min to evaporate the volatile hexane [15,22].

### 2.3. Design of System-on-a-Chip (SoC) Enabled Monitoring System

**Figure 4:**
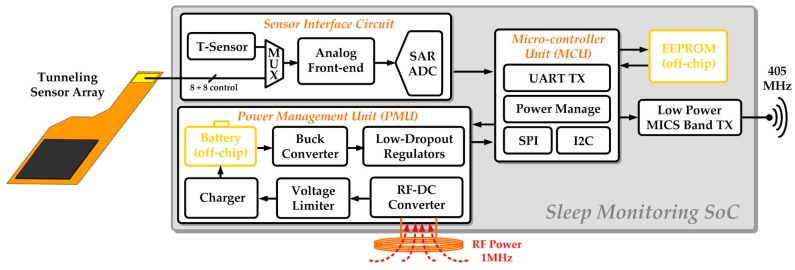
Block diagram of the proposed SoC enabled monitoring system.

The key feature of proposed SoC enabled monitoring system design is the intelligent detection of the tongue movement using a tunneling sensor array, wireless recharging and transmission to the cloud [16,23]. Figure 4 shows the block diagram of this system, which comprises of an assembled array of tunneling piezoresistive sensors, an analog front-end circuit (AFE), a 10-bit analog-to-digital converter (ADC), a power management unit (PMU), a digital micro-controller unit (MCU), a temperature sensor, and a low-power medical implant communication service (MICS) band transmitter.

The analog front-end circuit, which is designed for signal amplification and high signal-to-noise ratio (SNR) [24,25,26,27], operates in either pressure or temperature detection mode to deal with corresponding signals from the temperature or pressure sensors. Pressure-induced tunneling resistance variation is converted to voltage signals and linearly amplified in pressure detection mode. Temperature sensing is to sift out invalid data and prevent overheating due to abnormal situations. Successive-approximation-register (SAR) topology is chosen in ADC design to achieve ultra-low power consumption with a decent effective number of bits (ENOB) [28,29,30]. The wireless transmitter delivers the sensed physiological information to the wireless platform and then to the cloud. It operates at 405 MHz in MICS band to be compatible with general devices for medical applications, and on-off-keying (OOK) modulation is selected due to its simplicity and low power consumption [31].

For developing a reliable and convenient usage, a rechargeable button battery is used. Considering the module implementation, the battery can’t be large and thick so that its capacity is much limited. To sustain continuous monitoring of 8-h operation and control the system power budget, the power management unit, which consists of an RF-DC converter, a voltage limiter, a charger [32], an off-chip rechargeable button battery, a buck converter [33,34], and several low-dropout regulators (LDOs), is indispensable. The device is sealed in bio-compatible packaging for intraoral use, and thus is designed to harvest power wirelessly from the RF-DC converter, charging the battery through the on-chip charger. The buck converter is integrated to increase power transition efficiency. Each of the power domains in this system is allocated an LDO to ensure superior supply quality.

The digital MCU handles all the signals, and temporarily saves the data in the off-chip EEPROM before sending it to the MICS band transmitter for wireless transmission. In addition, MCU controls the timing of switching to perform the specific operation on demand such as the scanning of sensor array and also achieve power management incorporated with PMU. Essential I2C data format conversion is required to communicate with the off-chip EEPROM. Writing data into the EEPROM is very power-consuming, so it proceeds only when the data changes occur for better power efficiency and compact data storage.

### 2.4. Experiment Protocol

**Figure 5:**
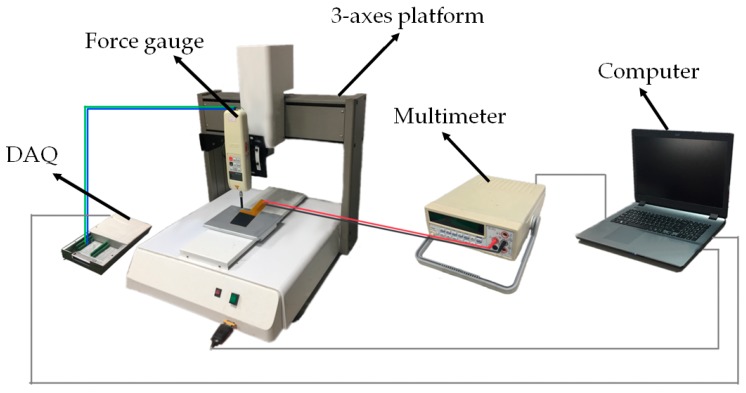
Experimental setup for testing the sensor array.

The key task of this system is to accurately detect the tongue position and record data on the timeline basis. The over time record of intraoral activities is indispensable to adjust physical structure of the device for fitting the requirement of each patient specifically [35,36]. First, the tunneling resistance of a stand-alone sensor array will be characterized by incremental forces. Figure 5 illustrates the experimental setup for the incremental force testing. A force gauge with a maximal resolution of 1 mN is used to deliver the applied force, where it is fixed on a 3-axes translational platform that has a vertical (Z-axis) displacement resolution of 1 μm. A circular cross-sectional rod of the force gauge presses the device under test, and the quantity of applied force is determined by the total vertical displacement. The resistance variation of each tunneling sensing cell is measured using a multimeter and recorded by a computer. Next, an actuator driven by a function generator will be utilized to give a periodical pressing for 7200 cycles in an hour to evaluate the dynamic response and repeatability of the sensor.

For the experiment of the proposed system, a prototype module of a compact size is implemented for demonstration with a graphical interface. Quantitative characterization of the applied force from a system point of view will be gone through once again using the identical setup shown in Figure 5. Subsequently, difference shapes of objects will be used to press the sensor array and examine the resolved forced images for the tongue position detecting demonstration. All the function blocks in SoC ship will be verified, and the measurement results of PMU and wireless transmitter are especially presented to confirm the capability of wireless charging and communication using the oscilloscope and spectrum analyzer.

## 3. Results and Discussion

### 3.1. Tunneling Piezoresistive Sensor Measurement

**Figure 6:**
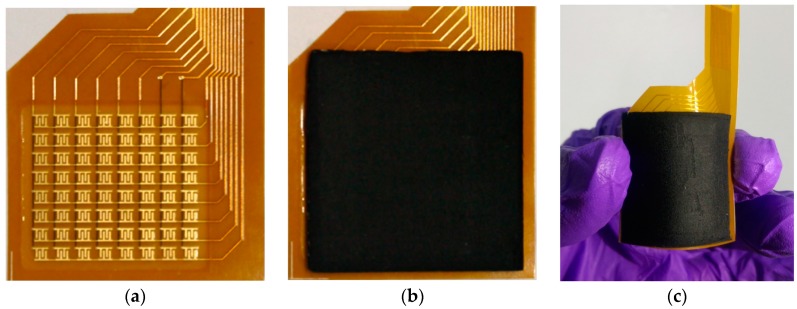
Sensor fabrication results: (**a**) Polyimide substrate layer with interdigitated electrodes; (**b**) Topped conductive polymer layer with interlocked microdome structures; (**c**) Flexibility of the assembled array.

An 8 × 8 array of tunneling sensors is assembled for the demonstration of this system. Figure 6a shows the polyimide substrate layer with identical interdigitated electrodes. The size of a single sensing cell is 1.8 × 1.6 mm^2^, and the gap between sensors is 2.2 and 2.65 mm horizontally and vertically. Figure 6b shows the topped conductive polymer layer with interlocked microdome structures which covers the whole 8 × 8 array of interdigitated electrodes. The assembled array of tunneling piezoresistive sensors and its flexibility is depicted in Figure 6c. Besides the ultra-high sensitivity under tiny pressure, the tunneling sensor has the advantages of simple fabrication process, easier integration, and good flexibility. For the system integration in a smart oral appliance, the prototype module is restricted to a very compact volume budget and also has to consider the camber surface inside the oral appliance. Thus this flexibility is much beneficial to the prototype module design.

**Figure 7:**
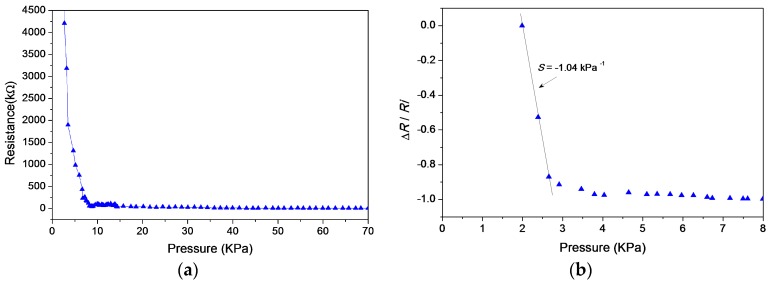
(**a**) Measured piezoresistive response of the tunneling sensor; (**b**) Normalized measured response under 8 kPa.

Figure 7a shows the measured piezoresistive response of the tunneling sensor. The applied force increases uniformly and monotonically to a sensing cell by the force gauge. The tunneling resistance reacts to tiny pressure (<3 kPa) because the piezoresistive effect is caused by the sharp variation of contact area, came from the rapid geometry deformation of interlocked microdomes. The sensitivity of tunneling resistance drops as the applied pressure exceeds 3 kPa. When the pressure is higher than 70 kPa, the resistance nearly stops decreasing, possibly because the total contact area reaches a steady value. Figure 7b shows the normalized measured response under 8 kPa. The resistance variation ΔR of the Y-axis is normalized by the initial resistance R_i_. The maximum of measured sensitivity (S = (ΔR/R_i_)/ΔP) marked in the figure is −1.04 kPa^−1^.The error bars of each data point stands for the measured minimum and maximum values.

**Figure 8:**
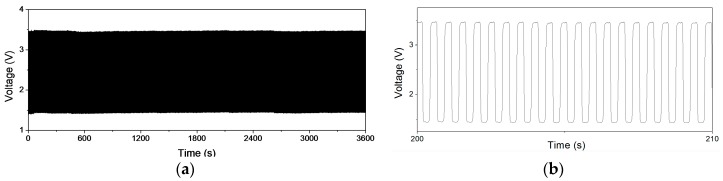
(**a**) Repeatability test for 7200 cycles at 2 Hz force pressing; (**b**) 20-cycles subset of (**a**) (200~210 s).

As shown in Figure 8a, the repeatability of a single sensing cell is verified by a measurement of dynamic transient responses for 7200 cycles. The sensing cell was repeatedly pressed using the piezoelectric actuator driven by a periodic square wave of 2 Hz, and the total duration of this measurement is one hour. Figure 8b is a 20-cycle subset of Figure 8a. This test utilizes a constant current source to convert the pressure-induced resistance variation to voltage signals. The raw data is recorded by a time step of 0.01 s and thus the whole test of 7200 cycles contains about 360,000 data points. The measurement results show that the repeatability of the fabricated sensing cells is quiet decent, where the cycle-to-cycle variation is approximately lower than 5%.

**Figure 9:**
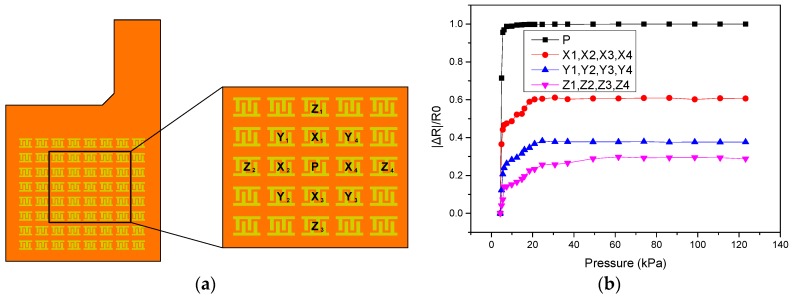
(**a**) Schematic of the proposed sensing array for the crosstalk study; (**b**) Measured resistance variation of the proposed sensing array.

Figure 9a shows a schematic for evaluating the crosstalk effect of the proposed sensing array. An external pressure force is applied to point P in this measurement, and the resistances at point P and the surrounding sensing elements points X, Y, and Z are measured independently. Figure 9b shows the measured results of resistance variation. When the external pressure applied at the point P grows, the resistance of point P decreases sharply. Because of the deformation at point P, the average resistance variation at the nearby points X, Y, and Z increases under zero applied pressure, and this crosstalk effect becomes weakened as the distance from point P increases.

### 3.2. Tongue Position Detectingprototype Module and Its Demonstration Results

**Figure 10:**
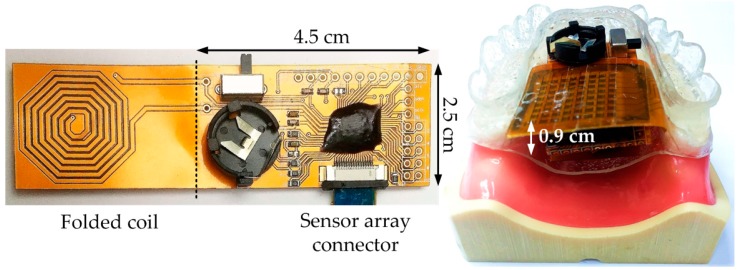
Prototype module for the demonstration of tongue position detecting.

In order to demonstrate the tongue position detecting function in a smart oral appliance, the prototype module (without EEPROM), which comprises a folded coil, a Li-ion rechargeable battery, a SoC silicon chip, the assembled array of tunneling sensors, and a few of on-board components, is implemented as shown in Figure 10. This module will be embedded on the roof of the oral appliance and sealed by a bio-compatible material (parylene in this case) for intraoral application. The size of assembled module is approximately 4.5 × 2.5 × 0.9 cm^3^, which just matches the available space in an oral appliance.

**Figure 11:**
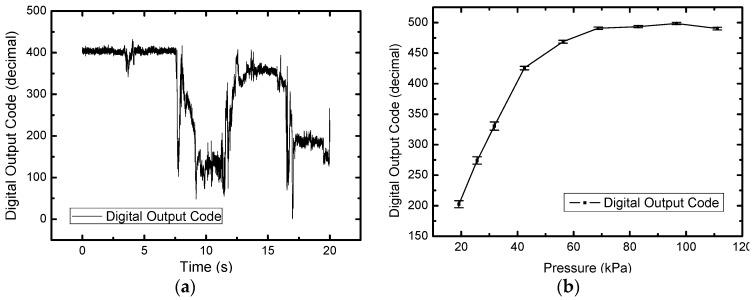
Measurement results of the prototype module in pressure detection: (**a**) Measured step transient response; (**b**) Measured characteristic of digital output code versus pressure.

Measurement results of the prototype module in pressure detection are exhibited in Figure 11. In Figure 11a, system step transient response is evaluated where four different forces are applied to the sensor cell sequentially by the force gauge. The result shows that the transient response of pressure sensing is fast (at millisecond level), and the digital output code rapidly settles to a steady value under one constant force. Figure 11b shows the measured characteristic of digital output code versus pressure. The characteristic presents an excellent linearity and sensitivity of 10 LSB/kPa from 20 to 45 kPa and starts approaching its steady value after 70 kPa. To be clear, 5 μm bio-compatible parylene is coated on the sensor array for the intraoral application in this demonstration. This coating slightly slows the dynamic response of the module and raises the pre-pressure to ~20 kPa comparing to a stand-alone tunneling sensor.

**Figure 12:**
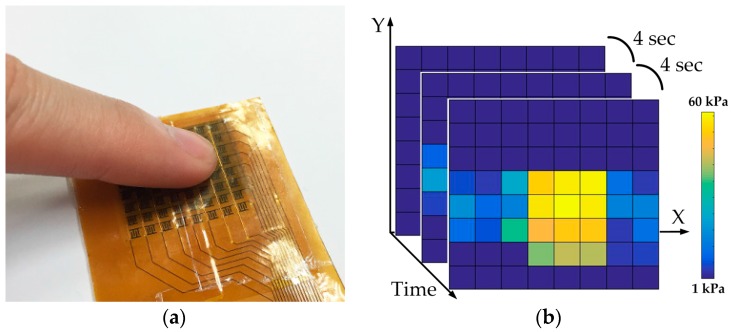
Tongue position detecting demonstration by the prototype module: (**a**) Fingertip pressing on the array; (**b**) corresponding force images recorded in continuous time frames using an interval of 4 s.

**Figure 13:**
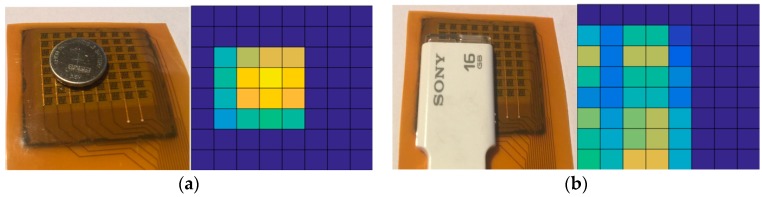
(**a**) Pressing test with a button battery and its corresponding force image; (**b**) Pressing test with a USB drive and its corresponding force image.

In the following measurement, tongue position detecting function is demonstrated by the prototype module connecting to a graphical interface to capture force images. Figure 12a shows a fingertip presses on the middle of sensor array with a mild force to simulate a tongue touch. Figure 12b illustrates the corresponding force images recorded in continuous time frames using an interval of 4 s. Each square in a force image stands for a signal sensing cell, and the brighter color in the square means the larger force pressing on the sensing cell. In Figure 12b, the shape of fingertip is clearly resolved by the sensing array. Furthermore, scanning force images every 4 s enables the smart oral appliance to position the tongue movement through the night sleep accurately by such kind of continuous-time monitoring. Next a button battery and a Universal Serial Bus (USB) drive are utilized to simulate different shapes of tongue contact in this demonstration. The force images shown in Figure 13a,b indicate that the shapes of objects under test are both successfully resolved by the sensing array.

### 3.3. Measurement Results of Wireless Charging and Communicationin the Proposed System

**Figure 14:**
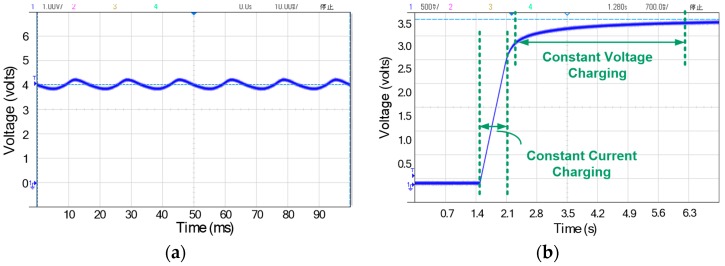
Measurement results of the power management unit: (**a**) Measured output waveform of the RF-DC converter; (**b**) Measured waveform of charging progress by the on-chip charger.

To achieve a total solution for OSA patients and fit their sustained use in daily lives, the smart oral appliance has to be capable of wireless charging and communication because of the overall bio-compatible coating. Figure 14 shows the measurement results of two significant checkpoints in the power management unit: the outputs of RF-DC converter and charger. When the device is placed on the wireless platform, 1 MHz RF wave of 5 V amplitude transmitted by the platform will be rectified to be around 4 V dc level by the on-chip RF-DC converter. In Figure 14a, the RF-DC converter delivers a measured dc voltage of 4.03 V with a little voltage ripple, which will be handled by the subsequent voltage limiter and regulator. To achieve rapid charging and simultaneously avoid the battery damage from overcharging, a charger with smooth control circuit is employed [28]. As the measured waveform of charging progress shown in Figure 14b, the Li-ion battery is first charged to about 3 V by a constant current (reconfigurable) and then smoothly switched to constant voltage charging to finish at 3.6 V. 

**Figure 15:**
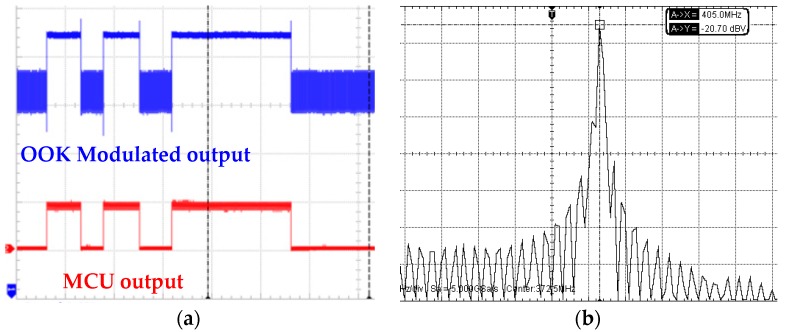
Measurement results of the MICS band transmitter: (**a**) Measured output waveforms of MCU and transmitter; (**b**) Measured transmitter output spectrum.

When the device harvests the RF power delivered by the wireless platform to charge the battery, wireless transmission is on duty as well. The operating frequency of wireless transmitter is selected as 405 MHz to meet the MICS band standard, and the frequency of RF signal for wireless charging is thus chosen as 1 MHz to avoid mutual interference. As the wireless platform is ready to retrieve physiological data, MCU reads the data from EEPROM and sends it to the transmitter sequentially. The transmitter will deliver the data wirelessly after OOK modulation. Figure 15a shows the measured output waveforms of MCU and transmitter. The physiological data from EEPROM is arranged sequentially in RS232 format by MCU and subsequently modulated by the transmitter. Figure 15b shows the measured transmitter output in spectrum view. The main tone is located at 405 MHz and output power is −7 dBm (−20 dBv).

**Figure 16:**
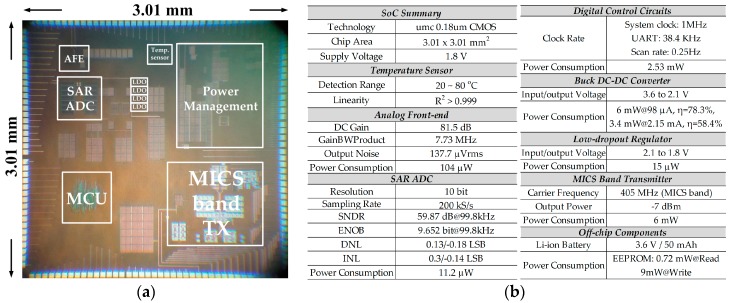
(**a**) The micrograph of the proposed SoC; (**b**) Measured performance summary.

This SoC chip, whose micrograph is shown in Figure 16a, is designed and implemented in a standard 0.18 μm CMOS process. The chip area is 3.01 × 3.01 mm^2^, including the testing pads. All the key functionalities of the proposed system have been verified according to the measurement results presented above. The measured performance summary in Figure 16b concludes the detail measurement results of all the sub-blocks in the proposed system.

## 4. Conclusions

Oral appliance therapy is effective and popular for OSA alleviation, but the time-consuming fitting progress reduces its efficiency. The proposed smart oral appliance provides continuous-time monitoring and physiological data collection of tongue movement through whole night sleep, which is significant for subsequent diagnoses and accelerates the fitting progress considerably. The prototype module has been implemented and sufficiently compact fitting in the available space of an oral appliance. The ultra-high sensitivity and capability of the tunneling sensor array to position the subtle pressure from tongue movement has been proven by well resolving the shapes of objects in the demonstration. The specifically designed SoC chip has also been fabricated and measured to provide multiple key functions and shrink the total module size and power consumption. This device is capable of harvesting external RF power for wireless charging, and the PMU inside will further improve the scheme of consumed power to guarantee an 8-straight-hour operation each night. The sensed physiological data can be retrieved by wireless transmission and connected with the big data and cloud-based applications.

This presentation would be the first of developing such a device capable of multiple intellectual functions to authors’ knowledge. Although this system has been preliminarily demonstrated with a prototype module in a sample oral appliance but not been experimented yet inside a real one on a patient, the following verification is definitely the next goal of this research. Using the proposed technique, this smart oral appliance could become a noninvasive, quiet, comfortable, low-cost, and portable solution, which benefits lots of OSA patients.
